# Orthopedic Trauma Management: A Comprehensive Review of Surgical Interventions, Rehabilitation, and Patient Outcomes

**DOI:** 10.7759/cureus.104429

**Published:** 2026-02-28

**Authors:** Naveenkumar Patil, Rahul Saket, Ravi Diwakar, G Harsha Vardhan Reddy, Kotteda Anil Kumar, Ashwin M Sathe

**Affiliations:** 1 Department of Orthopedics, KLE Jagadguru Gangadhar Mahaswamigalu Moorusavirmath Medical College and Hospital, HubbalIi, IND; 2 Department of Orthopedics, KLE Academy of Higher Education and Research, Deemed to be University, Belagavi, IND; 3 Department of Orthopedics, Hi-Tech Medical College, Rourkela, IND; 4 Department of Orthopedics, Virendra Kumar Sakhlecha Government Medical College, Neemuch, IND; 5 Department of Hepato-Pancreato-Biliary (HPB) Surgery and Liver Transplantation, Institute of Liver and Biliary Sciences, New Delhi, IND; 6 Department of Orthopedics, All India Institute of Medical Sciences (AIIMS) Mangalagiri, Mangalagiri, IND; 7 Department of Orthopedics, Jupiter Hospital, Pune, IND

**Keywords:** arthroplasty, fracture fixation, orthopedic trauma, rehabilitation, soft-tissue management

## Abstract

Orthopedic trauma remains a major global health challenge, with rising injury severity and increasingly complex fracture patterns demanding cohesive, evidence-based management. Despite advances in fixation technologies and rehabilitation science, clinical outcomes remain highly variable, largely due to inconsistencies in soft-tissue management, postoperative care, and the integration of emerging technologies. This narrative review synthesizes contemporary literature from the past decade to clarify current best practices and highlight persistent gaps across surgical stabilization, joint reconstruction, biological augmentation, rehabilitation strategies, and patient-specific determinants of healing. A comprehensive search of peer-reviewed sources was conducted to evaluate internal and external fixation methods, arthroplasty applications, soft-tissue reconstruction, rehabilitation protocols, and technological innovations, including 3D printing, computer-assisted navigation systems, and tele-rehabilitation platforms. Findings reveal that early, anatomically precise fixation combined with timely soft-tissue coverage and individualized rehabilitation significantly improves functional outcomes, while complications such as infection, nonunion, and implant failure continue to undermine recovery, particularly in patients with comorbidities or compromised bone quality. Technological advances show strong potential to enhance surgical precision, treatment personalization, and long-term functional outcomes, but remain insufficiently validated in large clinical populations. This review underscores the need for standardized treatment pathways, stronger multidisciplinary coordination, and more rigorous reporting of outcomes to optimize patient-centered recovery and guide future innovations in orthopedic trauma management.

## Introduction and background

Orthopedic trauma represents a significant clinical and population health issue due to its broad spectrum of injuries, including bones, joints, ligaments, and soft tissues, caused by both high-energy injuries such as road traffic collisions and low-energy falls, which have become more frequent in the population as they age [1,2]. As the fracture patterns have become more complex due to demographic changes and comorbidity problems such as osteoporosis, there has been a growing pressure on clinicians to apply sophisticated surgical techniques and structured programs of rehabilitation to ensure that the patient is optimally healed [3]. These trends emphasize the need for coordinated, timely, and evidence-based treatment plans that not only reverse the immediate injury but also minimize long-term disability [4].

The most effective orthopedic trauma treatment is multidisciplinary and holistic, grounded in the coordinated efforts of surgeons, anesthesiologists, physiotherapists, rehabilitation specialists, and nursing staff [5,6]. Recovery of anatomy, mechanical stability, effective pain management, and early functional recovery requires synchronization among the trauma management process, including prompt assessment, definitive fixation, and structured rehabilitation [7,8]. Although orthopedic trauma treatment has been modernized by advances in implants, imaging, and surgical methods, substantial variation is evident in clinical decision-making, outcome reporting, and rehabilitation procedures across institutions and regions [9,10]. The literature will therefore tend to provide fragmented information that does not reflect the full range of care required to ensure optimal recovery [11]. Moreover, factors such as age, nutritional status, comorbidities, and adherence to rehabilitation significantly influence patient outcomes; consequently, they are underrepresented or unmeasured in most studies [12,13].

Given these limitations, it is reasonable to propose an updated, integrated synthesis of current evidence to establish the relationships among surgical therapy, systemic rehabilitation, and long-term patient outcomes [14]. To be more specific, telerehabilitation can be defined as the remote provision of rehabilitation evaluation, monitoring, and guided care involving telecommunication tools [15]. Damage control orthopedics is the name of a stepwise approach to trauma, where temporary concussion control is undertaken to reduce physiological loads before permanent fixation is undertaken when the patient has been optimized for clinical purposes [16,17]. Studies that address these areas are relatively few. Still, the pattern of postoperative recovery is conditioned not only by the technical accuracy of the operations but also by the timeliness, speed, and quality of rehabilitation [18]. New technologies such as 3D-printed artificial implants, computer-assisted fixation, bio-enhanced healing materials, and less invasive interventions also require systematic assessment to determine their clinical efficacy and cost-effectiveness in real-life trauma patient groups [19,20]. The science of rehabilitation is also changing, and tele-rehabilitation platforms and AI-assisted monitoring devices are emerging as innovations that can expand access to care and improve adherence [21,22].

Objectives of the review

The objective of this review is to synthesize contemporary evidence on surgical interventions and rehabilitation strategies in orthopedic trauma to clarify how these components influence patient recovery. It aims to analyze outcomes across various fracture patterns and operative techniques and to identify the key clinical and patient-related factors that shape healing. Furthermore, the review seeks to highlight existing gaps and inconsistencies in current practice and propose evidence-based recommendations to enhance the overall quality and effectiveness of trauma care.

Methodology

The review employed a narrative design to synthesize up-to-date evidence on the management of orthopedic trauma by reviewing literature published from 2015 to 2025. The search was performed in PubMed, Scopus, Web of Science, and Google Scholar with the keywords "orthopaedic trauma", "fracture management", "surgical fixation", "external fixation", "internal fixation", "trauma rehabilitation", and "patient outcomes" using the database-specific Boolean operators. All records were consolidated, and duplicates were removed prior to screening. Relevance screening was conducted on titles and abstracts, and full-text screening was performed using preregistered eligibility criteria; reference lists of included articles were also screened to identify additional relevant studies. Eligible studies were peer-reviewed articles, clinical trials, cohort studies, systematic reviews, and meta-analyses of adult and geriatric populations that dealt with surgical methods, rehabilitation methods, or clinical outcomes. Cases, editorials, conference abstracts, non-English-language publications, and studies that compared pediatrics alone were excluded. Data about design, sample characteristics, type of trauma, surgical intervention, rehabilitation protocol, complications, and outcome measures (e.g., union rates, mobility, pain, and quality of life) were extracted and synthesized by thematic domain using a structured narrative approach; no formal risk-of-bias assessment or quantitative meta-analysis was conducted.

## Review

Epidemiology and classification of orthopedic trauma

Orthopedic trauma is a significant health problem worldwide, with a greater incidence rate in most parts due to urbanization, motor vehicle traffic, industrial growth, and aging of the population [23]. Communicable injuries: High-energy injuries, which are typical of road traffic accidents, industrial accidents, and high-impact sports, typically result in comminuted fractures and a high degree of soft-tissue injuries [24]. Conversely, the prevalence of low-energy falls at standing height among older adults is strongly associated with osteoporosis, and common fractures among them include hip, distal radial, and vertebral fractures [25]. The identification of these epidemiological variations aids risk stratification, pathway design, and the assessment of early and late complications [26].

Multiple systems are used to standardize descriptions, facilitate management, and enable comparable reporting [9]. The Arbeitsgemeinschaft für Osteosynthesefragen/Orthopaedic Trauma Association (AO/OTA) system is widely used for its anatomical location and fracture-morphology classification. It is commonly employed in clinical decision-making and comparative research [2]. The Gustilo-Anderson classification is commonly used to grade open fractures. It includes the variables of soft-tissue injury, contamination, and vascular compromise, which determine the urgency of surgery, the risk of infection, and the prognosis [18]. Specific systems (such as Neer for the proximal humerus and Garden for the neck of the femur) provide prognostic data on displacement, stability, and vascularity, assisting in operative planning and predicting the healing pattern [11]. The combination of these systems enhances diagnostic precision, interdisciplinary team communication, and research environments [24]. Table 1 indicates that fracture classification systems vary in their focus, criteria, and clinical use.

**Table 1:** Summary of major fracture classification systems used in orthopedic trauma AO/OTA: Arbeitsgemeinschaft für Osteosynthesefragen/Orthopaedic Trauma Association

Classification system	Anatomical/clinical focus	Key criteria	Clinical use/significance	Reference
AO/OTA classification	Long bones and major skeletal regions	Bone segment, fracture morphology, severity	Standardized communication, treatment planning, and research comparison	Salamanna et al. (2022) [2]
Gustilo-Anderson classification	Open fractures	Soft-tissue damage, contamination, vascular injury	Guides surgical urgency, infection risk, and soft-tissue coverage strategy	Suzuki et al. (2023) [18]
Neer classification	Proximal humerus fractures	Number of parts, displacement	Determines suitability for fixation vs arthroplasty; predicts functional outcome	Liang et al. (2024) [11]
Garden classification	Femoral neck fractures	Displacement, stability, vascular risk	Influences the choice between internal fixation and arthroplasty	Agostini et al. (2025) [14]

**Figure 1:**
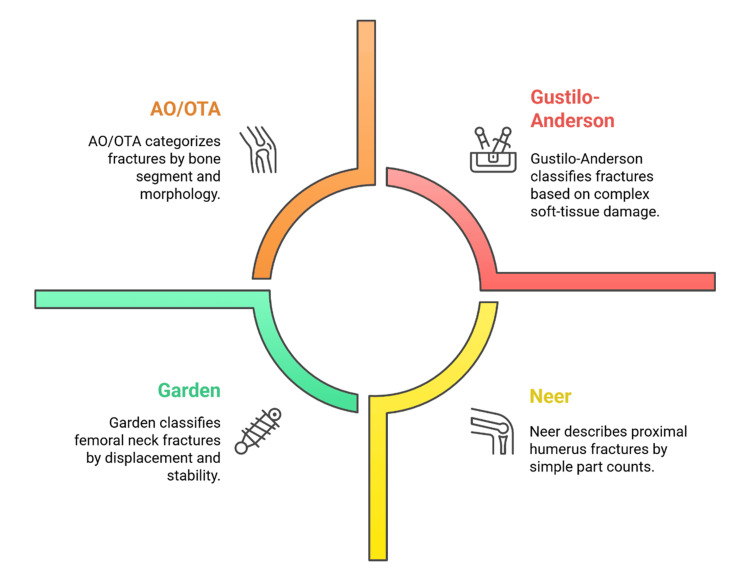
Major fracture classification systems in orthopedic trauma AO/OTA: Arbeitsgemeinschaft für Osteosynthesefragen/Orthopaedic Trauma Association Image Credit: Authors

Figure 1 shows the major outlines used to categorize orthopedic fractures.

Prehospital and emergency management of trauma

The ideal management of orthopedic trauma commences at the prehospital phase, where prompt intervention leads to less morbidity and facilitates prompt definitive treatment [27]. First responders use standardized systems, such as advanced trauma life support (ATLS), which address airway, breathing, circulation, disability, and exposure to manage life-threatening conditions in order of their relative importance for limb-related injuries [28]. Proper immobilization during transportation, particularly in cases of suspected spinal, long-bone, or pelvic injuries, reduces secondary tissue injury and promotes physiological stabilization [15]. During emergency department admission, clinicians perform primary and secondary surveys to detect musculoskeletal injuries and subsequently correct systemic derangements that may compromise interpretation [29]. Opioid analgesia, multimodal analgesia, and regional nerve blocks are ways to enhance patient comfort and prevent stress reactions leading to increased shock or metabolic instability in trauma patients [5].

**Figure 2:**
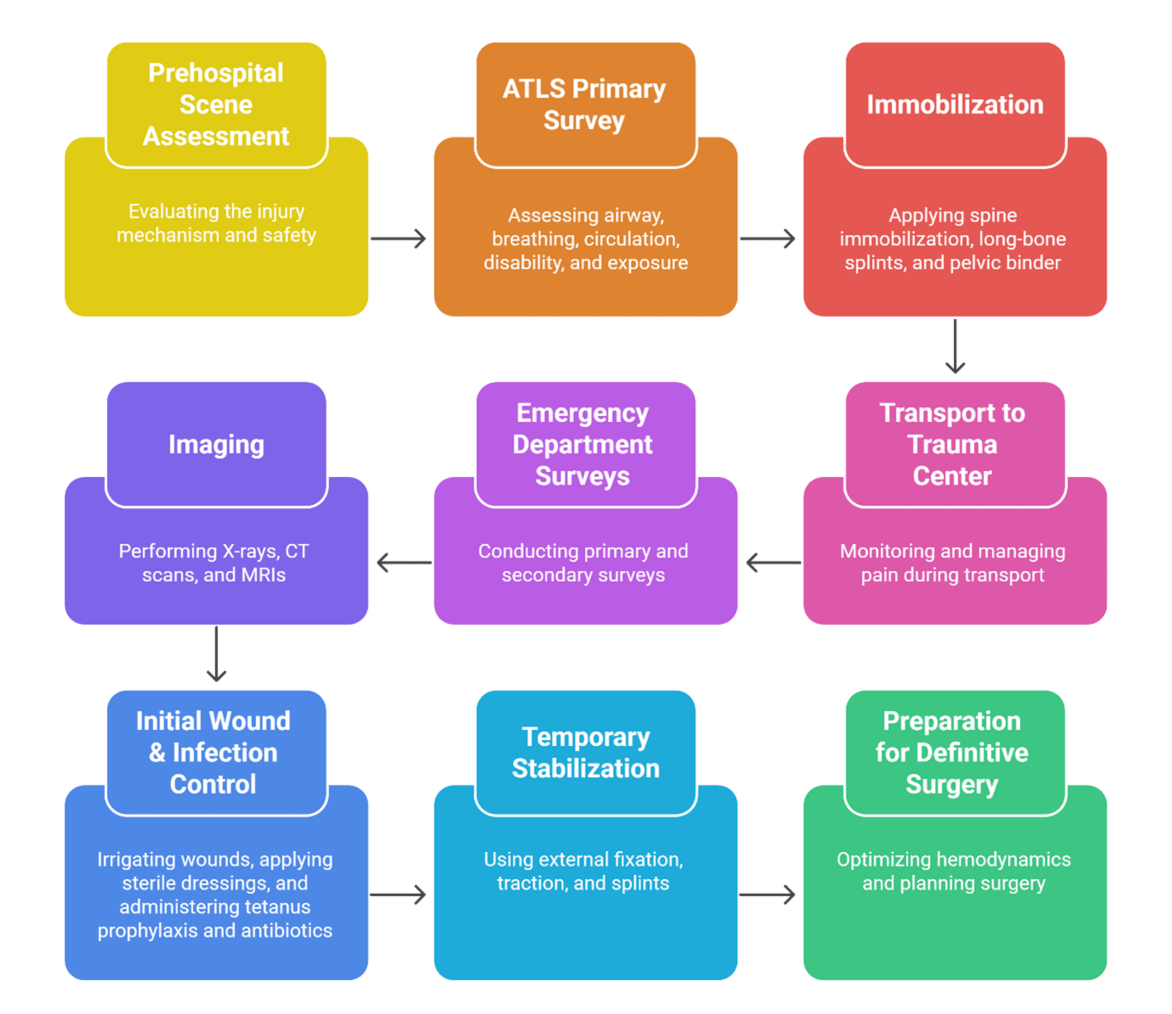
Pathway of early orthopedic trauma assessment and stabilization ATLS: advanced trauma life support, CT: computed tomography, MRI: magnetic resonance imaging Image Credit: Authors

The use of imaging improves the accuracy of early diagnostic methods; plain radiographs are often the first-line imaging modality, whereas CT/MRI is used when more information on fracture morphology, displacement, or soft-tissue involvement is needed [30]. Management of open fractures is initiated promptly with irrigation, sterile dressing, tetanus prophylaxis, and empiric antibiotics to reduce the risk of infection prior to surgical debridement [31]. Stabilization measures, including splints, pelvic binders, and external fixators, are used temporarily to maintain alignment, control hemorrhage, and facilitate safe mobilization until definitive surgical care becomes feasible [12]. The goals of coordinated emergency care include optimizing hemodynamic status, reducing early complications, and ensuring safe progression to operative management [32]. Figure 2 illustrates the step-by-step process for the prehospital and emergency management of orthopedic trauma.

Internal fixation techniques

The concept of internal fixation continues to dominate orthopedic trauma care, as it is the method by which alignment and stability are restored, with early mobilization permitted [33]. The choice of open reduction and internal fixation with plates, screws, or intramedullary devices is based on the location, pattern, and complexity of the fracture, particularly those fractures that are displaced or intra-articular and need anatomical reduction [7]. Stable fixation and controlled force transmission. Plate constructs, such as dynamic compression plates and locking compression plates, provide stable fixation with adjustable force transmission, particularly in metaphyseal or periarticular fractures and in compromised bone [21]. The locking plate technology enhances fixation reliability in osteoporotic bone by forming fixed-angle constructs that achieve fixation despite low cortical purchase [10]. Intramedullary nailing has been widely used to treat most diaphyseal long-bone fractures (femur, tibia, humerus), as it provides load-sharing stability with minimal disruption of soft tissues [34].

Screw-only fixation is typically used in selected designs, such as simple intra-articular fractures and avulsion injuries, where compression restores congruity and stability with minimal exposure [3]. Minimally invasive plate osteosynthesis has become one of the most popular methods because it preserves periosteal blood circulation and reduces iatrogenic injury to soft tissues while providing adequate mechanical stability to facilitate fracture healing [27]. Despite these developments, internal fixation carries risks, including infection, implant failure or loosening, malunion, and nonunion, particularly in patients with comorbidities and poor bone quality [14]. These risks underscore the importance of meticulous preoperative planning, adherence to biomechanical principles, and secure intraoperative technique to maximize outcomes and reduce the need for revision surgery [35]. Continued experience with implant materials, biologic adjuncts, and intraoperative imaging continues to sharpen internal fixation techniques and expand indications in complex trauma care [36].

External fixation methods

External fixation is a significant factor in orthopedic trauma, particularly when the patient requires urgent stabilization or when soft-tissue penetration precludes urgent internal fixation [37]. It may be used as a provisional or final measure, and the choice of the construct depends on the subject's physiology and the extent of the inflicted soft-tissue trauma [38]. The method fixes fractures with percutaneous pins, which are then connected to external rods or ring structures, without treating the fracture site directly [35]. This method is particularly applicable in high-energy trauma, open fractures, and polytrauma, where the swelling, contamination, or impaired vascularity may restrict internal fixation [8]. External fixation is essential, as it ensures limb alignment, reduces pain, and enables continued patient evaluation in cases of severe injury [39].

One of them is the rapidity of application in emergencies and limited-resource environments [14]. Damage-control orthopedics focuses on external fixation and, in hemodynamically unstable patients, emphasizes preventing physiological insults and reducing the incidence of complications, including fat embolism, acute respiratory distress syndrome, and systemic inflammatory response [5]. External fixation can be used in open fractures to facilitate repeated debridement and subsequent soft-tissue reconstruction with minimal additional damage to already injured tissues [22]. Though it can be employed as a temporizing step prior to conversion to internal fixation, in some cases it can be the final treatment, such as in tibial shaft fractures involving severe soft-tissue trauma, contamination, or poor access to high-technology internal fixation implants [40]. Its value is based on its modularity and the ability to integrate with staged protocols in a wide range of clinical settings [31]. Table 2 demonstrates that external fixation facilitates stabilization, wound management, and staged management across a variety of trauma cases.

**Table 2:** External fixation in orthopedic trauma: indications, mechanisms, advantages, and clinical applications ARDS: acute respiratory distress syndrome, SIRS: systemic inflammatory response syndrome

Aspect	Core description	Clinical purpose	Examples/applications	Reference
Rationale for use	Stabilizes fractures when soft tissue or physiology prevents internal fixation	Ensures early stabilization and reduces soft-tissue trauma	High-energy injuries; unstable polytrauma	Xames and Topcu (2024) [37]
Biomechanical principle	Percutaneous pins connected to rods or circular frames	Maintains alignment without exposing the fracture site	Unilateral fixators: ring (Ilizarov) fixators	Kushchayev et al. (2019) [28]
Primary indications	Severe soft-tissue injury, contamination, or swelling	Provides safe temporary stabilization	Open fractures; contaminated wounds	Kirven et al. (2020) [8]
Acute management advantages	Rapid application with minimal soft-tissue disruption	Pain reduction, alignment preservation, and initial fracture care	Emergency and resource-limited settings	Jung et al. (2024) [39]
Damage control role	Reduces physiological stress in unstable patients	Prevents fat embolism, ARDS, SIRS	Used before definitive fixation	Vincent et al. (2015) [5]
Wound management utility	Allows irrigation, antibiotic delivery, and repeated debridement	Facilitates soft-tissue reconstruction	Open tibial fractures needing staged care	Shivasabesan et al. (2018) [22]
Definitive treatment potential	Viable when internal fixation is unsafe or unavailable	Long-term fracture stabilization	Severe tibial shaft fractures	Prodinger and Taylor (2018) [40]
Practical features	Modular, adjustable, adaptable to swelling changes	Supports staged surgical protocols	Widely used in diverse trauma systems	Lex et al. (2023) [31]

Joint reconstruction and arthroplasty in trauma

Joint reconstruction and arthroplasty play a significant role in complex articular fractures, particularly in elderly patients or in fracture patterns in which internal fixation may not yield a stable and predictable result [41]. Such methods are typically used in joints with challenging fixation requirements, such as the hip, shoulder, and elbow, where comminution, bone quality, or vascularity is compromised, thereby reducing fixation success [19]. Hemiarthroplasty is commonly employed in older patients with displaced femoral neck fractures to provide pain relief and early mobilization, particularly when avascular necrosis or fixation risks are present [7]. Total hip arthroplasty may be associated with superior functioning and reduced reoperations in more active patients or those with known degenerative disease [33].

This is especially true in traumatic shoulder conditions, such as complex fractures of the proximal humerus with several fragments, where reverse shoulder arthroplasty is increasingly becoming the new form of surgery [12]. Elbow arthroplasty may be indicated in older patients with severely comminuted distal humerus fractures that are difficult to reconstruct reliably with internal fixation and are associated with a high risk of failure [28]. Arthroplasty is preferable to anatomical reconstruction when long-term, durable joint congruity is unlikely to be achieved or when early mobilization is needed to minimize long-term stiffness and functional deficits [42].

**Figure 3:**
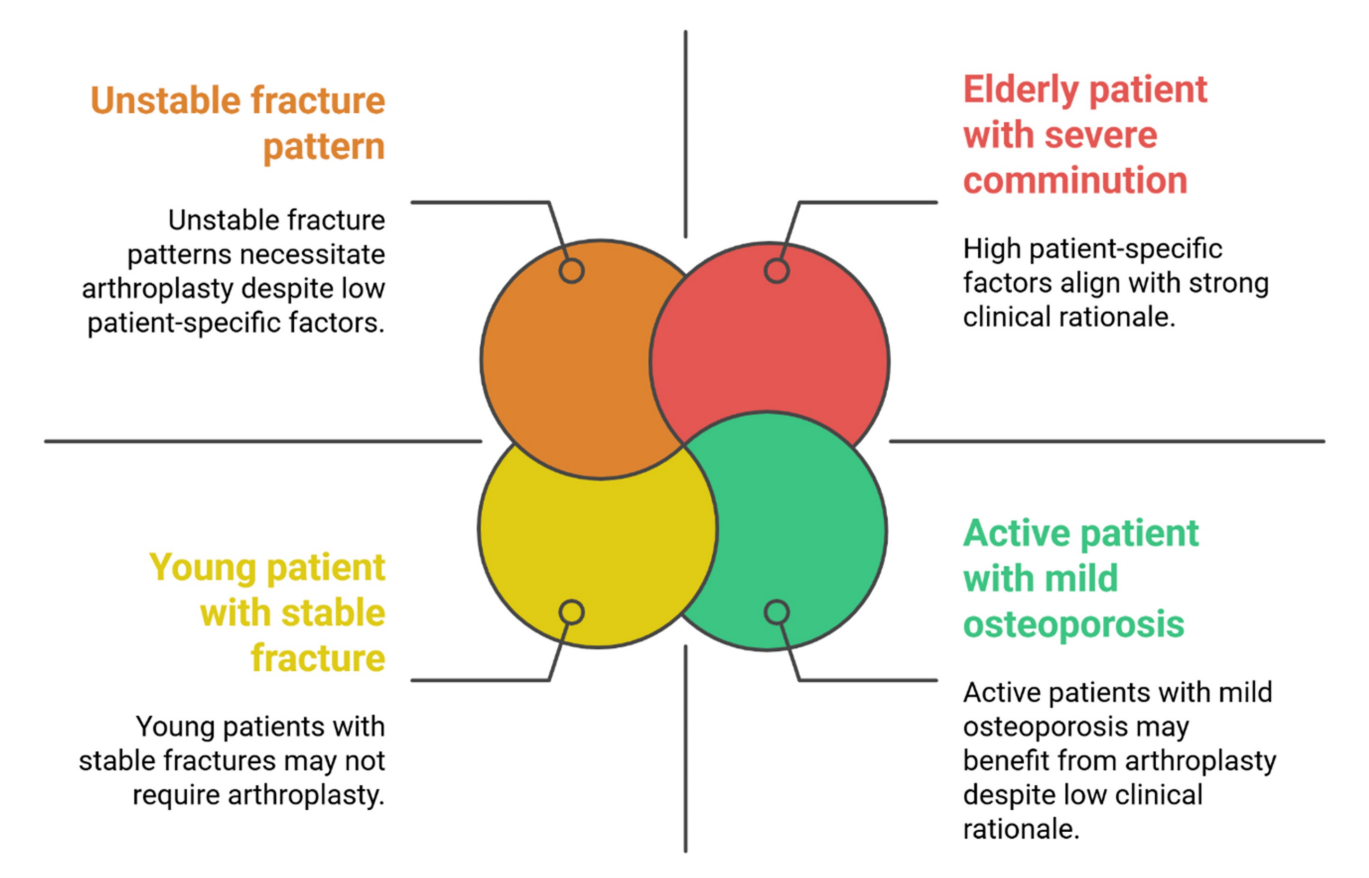
Determinants of arthroplasty selection in orthopedic trauma patients Image Credit: Authors

Despite the expected analgesic and functional advantages, arthroplasty has drawbacks, including concerns about implant longevity, dislocation, and limitations on postoperative activity [16]. Joint replacement is based on patient-specific factors, including age, comorbidities, functional demands, and bone quality [43]. There should be a balance between short-term stability and long-term durability in decision-making, particularly in younger patients, for whom revision surgery is technically challenging [44]. Arthroplasty in trauma is intended to stabilize joint function and enhance overall quality of life, rather than merely replace structural loss. Figure 3 demonstrates that several patient and clinical factors inform the choice of arthroplasty.

Soft-tissue management in orthopedic trauma

Soft-tissue management is a critical success factor in orthopedic trauma care because the quality of the surrounding skin, muscle, vessels, and nerves largely affects the timing of surgery, surgical safety, and functional outcomes [15]. The initial assessment is based on tissue viability, contamination, perfusion, and the presence of compartment syndrome, all of which inform immediate management decisions [13]. Debridement is a central pillar of open fracture treatment because it eliminates devitalized tissue and reduces the pathogen burden in the open wound, thereby promoting a healthier healing environment [30]. Staged and repeated debridement is, in most instances, necessary to result in an acceptable wound bed that would be suitable to close or reconstruct [22].

Wound coverage strategies vary widely depending on the size and extent of tissue damage; options may range from primary closure for minor damage to complex reconstructive approaches for large defects [11]. The rotational flaps and fasciocutaneous flaps are typically utilized when soft-tissue coverage is moderate. Still, when there are large defects or exposed bone and implants, free tissue transfer is necessary [45]. Early soft-tissue coverage, preferably within the first few days after injury, has been repeatedly associated with low infection rates, improved wound healing, and reduced nonunion rates [46]. Orthopedic and plastic surgery teams are closely associated in many cases of complex injuries, with coordination between fixation and coverage plans to achieve optimal results [12].

Ligamentous, neural, vascular, and tendon injuries are also frequently evaluated in soft-tissue management, as they are critical determinants of long-term limb function [34]. Timely repair of vascular injuries is crucial for limb salvage, and timely nerve repair may determine future sensory and motor recovery, depending on the type of nerve injury and its timing [18]. Infection control is a key issue in the treatment process and is facilitated by the timely use of antibiotics, sterile surgical practice, and organized wound care protocols [47]. Finally, effective soft-tissue care provides the biological basis for achieving stable fixation and enables efficient rehabilitation, underscoring its indispensable role in the overall treatment of orthopedic trauma [48].

Postoperative rehabilitation strategies

Management of orthopedic trauma has identified postoperative rehabilitation as a key determinant of successful outcomes, as functional recovery is determined not only by the quality of surgical fixation but also by the timing, progression, and suitability of rehabilitation programs [7]. The best approach is a structured, gradual process that begins with passive or assisted range-of-motion exercises to reduce stiffness, maintain joint mobility, and reduce postoperative edema [18]. In this early phase, attention is focused on ensuring the stability of the surgical construct, enhancing tissue healing, and preventing complications associated with excessive immobilization [49]. As radiographic evidence of fracture stability is revealed, patients proceed to active range-of-motion activities and progressive strengthening exercises to enhance muscle mass, neuromuscular coordination, and joint stability [12].

The final phase of rehabilitation focuses on functional retraining based on the patient's work-, life-, or sport-related requirements to facilitate a safe return to pre-injury activity levels [41]. Practice in biomechanical safety will always yield better results due to its ability to stimulate osteogenesis, increase synovial fluid distribution, and minimize the risk of contracture [20]. Delayed mobilization, by contrast, is associated with muscle atrophy, joint stiffness, and poorer long-term functional recovery [3]. Physiotherapists assist patients by individually guiding them through exercise programs, evaluating pain responses, and supervising safe progression through the stages of rehabilitation [46]. The occupational therapists augment this care by providing fine-motor coordination training, activities of daily living, adaptive techniques, and return-to-work preparation, particularly for upper-limb trauma [21]. Multimodal analgesia and, in certain cases, regional anesthesia are also useful and contribute to effective rehabilitation by facilitating patient involvement and minimizing the adverse effects of the therapy [15]. Finally, interdisciplinary care, patient-centered planning, and exercise progression, calculated based on healing milestones, are key to optimal recovery [50]. As shown in Table 3, rehabilitation incorporates phased exercises, functional training, and interdisciplinary support.

**Table 3:** Key components of postoperative rehabilitation in orthopedic trauma ROM: range of motion, ADL: activities of daily living, NSAIDs: nonsteroidal anti-inflammatory drugs

Rehabilitation phase/component	Core description	Clinical purpose	Examples/applications	Reference
Early phase rehabilitation	Passive or assisted range-of-motion exercises	Minimize stiffness, maintain joint mobility, reduce oedema	Gentle ROM, assisted mobilization	Suzuki et al. (20230 [18]
Protection of surgical construct	Controlled movement to protect fixation	Prevent implant stress and support tissue healing	Restricted weight-bearing, bracing	Costa et al. (2017) [49]
Progressive strengthening phase	Advancement once radiographic stability appears	Improve muscle mass, coordination, and joint stability	Active ROM, isotonic exercises	Kaye et al. (2019) [12]
Functional retraining stage	Task-specific rehabilitation tailored to patient needs	Enable safe return to daily activities, work, or sport	Gait training, occupational task practice	Barile et al. (2024) [41]
Benefits of early mobilization	Mobilization when biomechanically safe	Enhances osteogenesis and reduces stiffness and contractures	Early weight-bearing protocols	Gilboa et al. (2019) [50]
Risks of delayed mobilization	Immobilization beyond the healing window	Muscle atrophy, joint stiffness, reduced long-term function	Prolonged immobilization post-fracture	Alsawair et al. (2024) [3]
Physiotherapy role	Guides exercise progressions and monitors pain	Ensures safe rehabilitation and prevents complications	Supervised strengthening, ROM monitoring	Twersky et al. (2024) [46]
Occupational therapy role	Supports fine motor skills and daily activity adaptation	Facilitates return-to-work readiness and independence	ADL training, upper-limb functional tasks	Chen et al. (2023) [21]
Pain management support	Multimodal analgesia and regional anesthesia	Improves patient participation and reduces therapy pain	NSAIDs, nerve blocks, analgesic combinations	Testa et al. (2021) [15]
Interdisciplinary coordination	Collaborative planning across healthcare teams	Ensures tailored, safe, and progressive recovery	Multi-speciality rehab rounds	Zhou et al. (2024) [43]

Complications in orthopedic trauma management

Complications in orthopedic trauma remain a major clinical concern due to their potential to impede healing, prolong disability, and increase reoperation rates [9]. One of the most critical complications is nonunion and malunion, which can result from insufficient stability, poor vascularity, infection, metabolic disorders, or mechanical overload [32]. Nonunion can be hypertrophic or atrophic and may require different management strategies; malunion can result in deformity, pain, gait abnormalities, and premature degenerative joint disease [43]. Another severe complication is infection, especially with open fractures or when the soft tissues are so severely disrupted, which can jeopardize the functionality of implants or even require aggressive debridement [45].

Complications associated with implants, such as mechanical failure, fatigue fracture, or loosening, arise when fixation constructs are subjected to excessive mechanical forces or when bone quality is insufficient to maintain implant stability [8]. Trauma patients are also at high risk of venous thromboembolism owing to the sustained immobilization status, the inflammatory state of the organism, and hypercoagulable conditions [25]. Compartment syndrome is a limb-threatening emergency that may cause muscle necrosis or nerve damage that may be irreversible unless treated in a timely manner [19]. Even following radiographic healing, chronic pain syndromes, including complex regional pain syndrome, can occur and lead to a significant source of long-term disability [2].

Several patient-associated variables are predictive of an elevated risk of complications. Older age, osteoporosis, diabetes, peripheral vascular disease, and immunosuppression impair healing and predispose to infection [37]. Surgical lag time, due to medical or logistical factors, can delay alignment, increase tissue softness, and increase the incidence of complications [14]. The most important aspect of successful management is close attention to the postoperative period, the ability to recognize warning signs, adherence to evidence-based recommendations, and close patient education to facilitate timely reporting and treatment adherence [23].

Influence of patient factors on outcomes

Personal factors have a strong impact on both biological recovery and functional outcome following orthopedic trauma [6]. The age-related determinant is among the most critical, as young patients exhibit more rapid bone remodeling and better rehabilitation tolerance than older patients, who are frequently challenged by frailty, reduced vascularity, and osteoporotic bone [10]. Bone quality is especially important; osteoporotic bone poses challenges for surgical fixation, as it provides less screw purchase and is more likely to fail under mechanical stress [13].

Another important component of healing is nutritional status, as deficiencies of protein, vitamin D, calcium, and other essential micronutrients impair callus formation and increase the risk of delayed union or infection [20]. In other comorbidities, including diabetes, renal failure, smoking, and peripheral vascular disease, dysfunctional perfusion, modified inflammatory reactions, and worse outcomes are noted [11]. Psychological variables such as anxiety, depression, fear of reinjury, and lack of self-efficacy may decrease rehabilitation participation and adversely affect the perception of pain [28]. Social support, motivation, and psychological resilience, on the other hand, are associated with better adherence and functional recovery [31].

Lifestyle behaviors further determine outcomes. Smoking has been closely linked to a slow union and wound healing and is highly correlated with higher infection rates because of its vasoconstrictive and tissue-hypoxic properties [16]. High levels of alcohol interfere with the metabolism of the bones and decrease their adherence to rehabilitation [27]. Recovery is also affected by pre-injury fitness and activity levels, with patients who are more fit at baseline (both in cardiovascular fitness and body strength) recovering more quickly [18]. Identification of these personal variables will guide clinicians in individualizing treatment plans, anticipating difficulties, and implementing specific interventions, such as smoking cessation, nutritional supplementation, or psychological support, to maximize recovery [49].

Technological advances in trauma management

Technological innovation continues to advance orthopedic trauma care, offering improved precision, personalization, and biological healing potential [24]. 3D printing is increasingly used to model patient-specific anatomies, improving preoperative planning and the design of custom implants to address complex fractures [35]. Computer-assisted navigation and surgery also enhance accuracy during surgery by providing real-time spatial information for implant placement, improved alignment accuracy, and mechanical stability [4]. These benefits are extended to robotic-assisted systems, which enable highly reproducible implementation of surgical plans and reduce soft-tissue disturbance [30].

Biomaterials have also enabled biodegradable implants that provide temporary fixation and resorb slowly as the bone heals, eliminating the need for hardware removal [26]. Osteogenesis is activated by biological agents such as bone morphogenetic proteins, which are particularly effective in high-risk fractures or nonunions that have already occurred [43]. Ceramics, demineralized bone matrix, and synthetic scaffolds are bone graft substitutes that provide alternatives when an autograft is unavailable or impractical (e.g., due to donor-site morbidity) [1].

Rehabilitation has also taken on a new form with advances in technology. Tele-rehabilitation systems enable remote monitoring, real-time exercise instructions, and greater patient access in rural or underserved areas [36]. Wearables can continuously monitor joint mobility, limb loading, and gait patterns, providing clinicians with objective data to track rehabilitation progress more accurately [22]. AI can be used to predict the healing process, determine whether a person is at high or low risk of complications, and plan individualized treatment pathways [48]. As these technologies continue to advance, their integration will optimize decision-making, reduce complexity, and improve patient outcomes across orthopedic trauma care [47].

## Conclusions

This narrative review highlights the complexity and multidisciplinary nature of orthopedic trauma treatment, in which success depends on the coordinated integration of accurate surgical approaches, prompt soft-tissue treatment, well-organized rehabilitation, and consideration of patient-specific factors that influence the healing process. The review of existing evidence indicates that, with advances in fixation technologies, biologics, and digital technologies, the treatment approach is evolving to enable greater accuracy, personalization, and early functional restoration. Meanwhile, persistent issues, such as heterogeneous clinical procedures, variation in complication risk, and inconsistent reporting of outcomes, continue to limit the generalizability and comparability of the research results. New technologies such as computer-assisted surgery, 3D-printed guides, and tele-rehabilitation platforms have significant potential but require confirmation through high-quality, long-term studies. Altogether, this review supports the necessity of standard care processes, enhanced interdisciplinary collaboration, and larger-scale clinical trials to streamline patient-centered recovery. With advances in technology and the adoption of evidence-based practice, future orthopedic trauma management will enable more predictable healing, lower complication rates, and improved functional outcomes.
